# Periprocedural and clinical outcomes of percutaneous coronary intervention of chronic total occlusions in patients with low- and mid-range ejection fractions

**DOI:** 10.1186/s43044-020-00065-1

**Published:** 2020-05-24

**Authors:** Waleed Salem El Awady, Mohamed Samy, Mohammad Mustafa Al-Daydamony, Magdy Mohammad Abd El Samei, Khaled Abd El Azim Shokry

**Affiliations:** 1 Cardiology Department, Faculty of Medicine, Zagazig University, Zagazig, Ash Sharkia Egypt; 2 Cardiology Department, Military Medical Academy, Cairo, Egypt

**Keywords:** Chronic total occlusion, Percutanous coronary intervention, Left ventricular ejection fraction

## Abstract

**Background:**

The benefit and safety of percutaneous coronary intervention (PCI) to chronic total occlusions (CTO) in patients with low and mid-range left ventricular ejection fraction (LVEF) continue to be evidence limited. The aim of our study was to investigate the impact of LVEF in patients undergoing CTO PCI and to evaluate the mid-term clinical outcome of those with low and mid-range LVEF. We assessed the periprocedural and mid-term outcomes of 75 patients undergoing CTO PCIs according to pre-procedural LVEF: group (N), ≥ 50% (normal, nom.= 25); group (M), 40-49% (mid-range, nom.= 25); and group (L), < 40% (low, nom.= 25); within 6 months of follow-up.

**Results:**

The prevalence of DM and chronic kidney disease (CKD) was significantly higher in low LVEF group (60%, *p* = 0.04 and 48%, *p* = 0.01 respectively). Apart from significantly lower contrast volume in patients with low LVEF (*p* = 0.04), there was no significant difference between the three groups regarding the procedural time, SYNTAX score and J-CTO score. We noticed similar procedural success in the three groups (88% vs. 84% vs. 76%, *p* = 0.521). LVEF category failed to predict procedural success (OR = 0.652, *p* = 0.268). There was a highly significant improvement in angina 6 months following intervention in normal LVEF group (*p* value < 0.001). Grade of dyspnea significantly improved 6 months following intervention in mid-range LVEF and low LVEF groups (*p* value = 0.04 and 0.031 respectively). There was no significant difference between the three groups regarding the reported MACCEs (12% vs. 16% vs. 28%, *p* = 0.268).

**Conclusion:**

CTO PCI represents an efficient and safe strategy in patients with low and mid-range LVEFs. Mid-term outcomes in these patients were significantly improved following successful CTO PCI, without higher risk of MACCE at 6 months follow-up.

## Background

Coronary chronic total occlusions (CTOs) are described as an occluded coronary artery with thrombolysis in myocardial infarction (TIMI) flow 0 for a period exceeding 3 months. Euro CTO club consensus stratified the obstruction duration into 3 grades: (I) “certain” (confirmed by angiography), if TIMI 0 flow was confirmed by an angiogram done more than 3 months ago; (II) “likely” (confirmed by clinical data), if an acute myocardial infarction (MI) occurred more than 3 months ago in the occluded artery territory with no other culprits incriminated in the current angiography; (III) “undetermined”, TIMI 0 flow and angiographically anatomical configuration suggesting long-standing occlusion with stable angina unchanged in the last 3 months or evidence of silent ischemia [1].

CTOs are one of the most challenging targets of percutaneous coronary intervention (PCI), due to their complexity, with lower procedural success rates and greater complication rates, radiation exposure and procedure times compared with non-CTO PCI. Indeed, in stable multivessel coronary artery disease (CAD), CTO was the most robust independent predictor of failed PCI, and was associated with worse prognosis [2]. However, recent advances in the interventional techniques, dedicated equipment and CTO expertise have resulted in > 90% procedural success rates, and lower major complications [3]. Reported benefits of successful CTO PCI included improved symptoms, ventricular function, and survival [4].

Left ventricular ejection fraction (LVEF) is a well established independent prognostic factor in patients with CAD [5]. Severe ischemic left ventricular dysfunction is related to higher morbidity, an increased risk of sudden death due to ventricular arrhythmias, poor quality of life, and frequent re-hospitalization for heart failure, with CTO patients having more comorbidities and procedural drawbacks [6].

Data regarding procedural success and clinical outcomes of CTO PCI is limited throughout literature, in patients with low (< 40%) and mid-range LVEF (40-49%). The aim of our study was to assess the impact of LVEF in patients undergoing CTO PCI and to evaluate the mid-term clinical outcome of those with low and mid-range LVEF.

## Methods

### Study design

This prospective study was accomplished from November (2017) to April (2019). We included 75 patients coming to the catheterization laboratory unit to undergo elective CTO PCI.

The procedure was well explained to each patient and a detailed informed verbal consent was obtained. The study protocol was approved by our Institutional Clinical Research and Ethics Committee.

### Patient selection

Selected patients had at least one epicardial coronary artery CTO proved by former coronary angiography and indicated for PCI; i.e., presence of viable myocardium in the supplied territory in the presence of significant ischemia and/or presence of refractory angina {Class III/IV} or new crescendo angina not explained by any other lesions [7, 8].

We excluded patients with acute or recent coronary occlusions, recent revascularization in the past 3 months, non-viable myocardium, previous failure of CTO PCI, poor echocardiographic window, significant structural heart disease, decompensated heart failure, and active infections.

We subdivided the study population into 3 groups based on LVEF: group (N), normal LVEF (≥ 50%); group (M), mid-range LVEF (40-49%); and group (L), low LVEF (< 40%).

Every patient was assessed by full history analysis and thorough clinical examination. Laboratory analysis of serum creatinine, urea, liver function test, fasting and postprandial blood glucose, and lipid profile was performed. Twelve-lead ECG was assessed for evidence of ischemic heart disease (IHD) or prior MI. Baseline angina and dyspnea were graded according to Canadian Cardiovascular Society (CCS) [9] and New York Heart Association (NYHA) classes [10] respectively, prior to the CTO PCI, then reassesed 6 months post-PCI.

### Echocardiography

Every patient underwent echocardiography within 24 h before intervention, based on the recommendation of the American Society of Echocardiography [11, 12]. The echocardiographic examination was accomplished using General Electric Vivid 7 (Norway) and Siemens Acuson NC 1000 (Germany) machines, using 2 to 2.5 MHZ transthoracic transducers. Echocardiograms were performed in the supine and/or left lateral position. Modified biplane Simpson’s method was used to assess LVEF [11]*.*

Patients with impaired LVEF and without significant angina and/or new crescendo angina underwent low-dose dobutamine stress echocardiography (DSE) for assessment of contractile reserve. Hypokinetic or akinetic myocardial segments that showed improved contractility with low-dose DSE were considered viable [12].

### Coronary intervention

We used General Electric and SIEMENS catheterization systems for our procedures. CTOs were defined as an evidence of total occlusions in a major epicardial coronary artery (≥ 2.5 mm in diameter), with durations ≥ 3 months [1]. CTO lesions complexity and CTO PCI attempt difficulty were evaluated based on the J-CTO (Japanese multicenter registry) score [13]. Syntax score was used to evaluate the coronary lesions [14]. Coronary angioplasty was done for CTO lesions; with access site, techniques of wiring, and dedicated tools left for operators’ preferences. Interventions resulting in reduction of luminal diameter of less than 20% compared to the adjacent normal coronary vessel were considered successful. Interventions that could not achieve at least TIMI 2 flow were considered failed [15, 16]. Patients were prescribed guideline-derived optimal medical treatment, and a follow-up for 6 months was done.

### Clinical outcome and follow up endpoints

Procedural success was defined as angiographically successful revascularization without procedure-related complications, including cardiac death, acute MI, perforation with or without tamponade, access site complications, and contrast-induced nephropathy (CIN). Major bleeding was assessed according to TIMI bleeding classification [17]. Major adverse cardiac and cerebrovascular events (MACCE) were defined as the composite of cardiac death, MI, acute heart failure, stroke, CTO target vessel revascularization (TVR) and non-TVR, and assessed within 6 months of follow-up post CTO PCI.

### Statistical analysis

Data collected throughout history, basic clinical characteristics, and outcome measures; were coded and entered using the Microsoft Excel software, then imported into the Statistical Package for the Social Sciences (SPSS version 20.0) software for analysis. Chi-square and Fisher exact tests were used to compare categorical data. Continuous data were summarized as mean ± standard deviation (SD). One way ANOVA test was used for comparison of LVEF in the three groups. Paired sample *t* test was performed to assess the difference between means ± SDs of one variable before and after intervention. Finally, independent predictors for procedural success were identified using univariate and multivariate binary logistic regression analyses models.

## Results

The study population had a mean age of 60.51 ± 8.09 years and a mean BMI of 27.68 ± 2.1. 60 patients (80%) were males, 58 patients (77.3 %) had hypertension, 47 patients (62.7%) had dyslipidemia, 53 patients (70.7%) were current smokers, while CKD was recorded in 20 patients (26.7%).

Group (L) showed a significantly higher prevalence of CKD and DM (*p* < 0.05). However, the 3 study groups showed no significant difference as regard the age, BMI, dyslipidemia, gender, hypertension, history of previous revascularization, or smoking (*p* > 0.05) (Table 1). Clinically, patients with advanced exertional chest pain of CCS class ≥ 3 were significantly higher in group (N) (*χ*^2^ = 9.301, *p* < 0.05), while exertional dyspnea of NYHA class ≥ 3 was significantly higher in group (L) (*χ*^2^ = 12.052, *p* < 0.05) (Table 2).

**Table 1:** Patient characteristics and risk factors in the study groups

Variable	G (N) (nom. = 25)	G (M) (nom. = 25)	G (L) (nom. = 25)	*p* value
Age (years) (mean ± SD)	60.40 ± 8.37	60.042 ± 7.91	61.07 ± 8.24	0.901
BMI (kg/m^2^) (mean ± SD)	27.69 ± 2.44	27.81 ± 2.21	27.58 ± 1.64	0.928
CKD (nom. (%))	3 (12%)	5 (20%)	12 (48%)	0.01*
DM (nom. (%))	7 (28%)	8 (32%)	15 (60%)	0.042*
Dyslipidemia (nom. (%))	14 (56%)	16 (64%)	17 (68%)	0.671
Hypertension (nom. (%))	20 (80%)	18 (72%)	20 (80%)	0.738
Male (nom. (%))	21 (84%)	20 (80%)	19 (76%)	0.779
Post CABG (nom. (%))	3 (12%)	5 (20%)	4 (16%)	0.743
Prior PCI (nom. (%))	8 (32%)	10 (40%)	5 (20%)	0.304
Smoking (nom. (%))	16 (64%)	17 (68%)	20 (80%)	0.433

Data are expressed as mean ± SD or number (%)

*BMI*: body mass index, *CABG*: coronary artery bypass graft, *CKD*: chronic kidney disease, *DM*: diabetes mellitus, *G* (*N*): normal LVEF group, *G* (*M*): mid-range LVEF group, *G* (*L*): low LVEF group, *:significant

**Table 2:** Baseline clinical data in the 3 groups

Variable	G (N) (nom. = 25)	G (M) (nom. = 25)	G (L) (nom. = 25)	*p* value
	Nom. (%)	Nom. (%)	Nom. (%)	
CCS class				
1, 2 3, 4	3 (12%) 22 (88%)	10 (40%) 15 (60%)	13 (52%) 12 (48%)	0.01*
NYHA class				
1, 2 3, 4	19 (76%) 6 (24%)	15 (60%) 10 (40%)	7 (28%) 18 (72%)	0.002*

Data are expressed as numbers (%)

*CCS*: Canadian Cardiovascular Society; *G* (*N*): normal LVEF group, *G* (*M*): mid-range LVEF group, *G* (*L*): low LVEF group; *NYHA*: New York Heart Association, *:significant

Apart from contrast volume that was significantly lower in patients with low LVEF (F = 2.95, p < 0.05), there was no significant difference between the three groups regarding the procedural time, SYNTAX score, and J-CTO score. Antegrade wiring technique was successful in 57 (76%) out of the 75 patients, with no statistically significant difference among the three groups (p < 0.05). Guidewires of < 3 gm tip load were used in 48 (64%) out of 75 patients, with almost same percentage among the three groups (p > 0.05) (Table 3).

**Table 3:** Angiographic characteristics and procedural data in the 3 groups

Variable	G (N) (nom. = 25)	G (M) (nom. = 25)	G (L) (nom. = 25)	*p* value
Procedure time (min)				
Mean ± SD	79.12 ± 30.24	80.36 ± 27.54	80.28 ± 25.32	0.985
Range	35-150	36-145	40-130	
Contrast volume (ml)				
Mean ± SD	357.3 ± 215.2	339.8 ± 198.4	276.4 ± 139.4	0.04*
Range	142-573	141-538	137-415	
Syntax score				
Mean ± SD	21.64 ± 3.90	22.72 ± 3.35	22.84 ± 2.56	0.376
Range	15-30	17-29	19-29	
J-CTO score (N. (%))				
≥ 3	12 (48%)	13 (52%)	15 (60%)	0.687
< 3	13 (52%)	12 (48%)	10 (40%)	
Wire technique				
Antegrade	20 (80%)	19 (76%	18 (72%)	0.803
Retrograde	5 (20%)	6 (24%)	7 (28%)	
Wire stiffness				
< 3 g	17 (68%)	15 (60%)	16 (64%)	0.841
> 3 g	8 (32%)	10 (40%)	9 (36%)	

Data are expressed as mean ± SD or number (%)

*G* (*N*): normal LVEF group, *G* (*M*): mid-range LVEF group, *G* (*L*): low LVEF group, *:significant

Procedural success was recorded in 22 (88%) of 25 patients of group (N), while periprocedural complications were determined in 3 cases (1 case of NSTE-ACS, 1 case of non-flow limiting dissection and 1 case of self-limited coronary perforation without tamponade). In group (M), procedural success was recorded in 21 (84%) of 25 patients, while periprocedural complications were determined in 4 cases (1 case of AF, 1 case of heart failure, 1 case of coronary perforation with tamponade, and 1 case of non-major vascular site complication). In group (L), procedural success was recorded in 19 (76%) of 25 patients, while periprocedural complications were determined in 6 cases (3 cases of CIN, 2 cases of heart failure, and 1 case of AF). There was no significant difference in the procedural success between the three groups (*χ*^2^ = 1.303 and *p* > 0.05).

By applying univariate then multivariate logistic regression analysis models, only DM and J-CTO scores were proved to be independent predictors of procedural success; where periprocedural complications were more reported in diabetic patients and those with J-CTO score > 3 (OR = 4.884 and 15.882 respectively, *p* < 0.05). All other factors, including LVEF category failed to predict procedural success (Table 4).

**Table 4:** Univariate and multivariate logistic regression analyses for predictors of procedural success

Variable	Exp (B) odds ratio	95% CI	*p* value	Variable	Exp (B) odds ratio	95% CI	*p* value
Univariate logistic regression analysis							
DM	4.393	1.209/15.957	0.025*	Prior PCI	1.528	0.440/5.304	0.505
EF category	0.652	0.305/1.389	0.268	CKD	0.444	0.089/2.209	0.322
Age	1.055	0.975/1.142	0.182	Post CABG	1.767	0.406/7.692	0.448
Gender	0.485	0.126/1.865	0.292	SYNTAX score	0.905	0.756/1.084	0.279
BMI	0.909	0.689/1.197	0.496	J-CTO score	14.571	1.784/119.043	0.012*
HTN	0.384	0.106/1.385	0.144	Wiring technique	0.658	0.175/2.456	0.532
Smoking	0.604	0.173/2.107	0.429	Wiring stiffness	1.327	0.367/4.799	0.666
Dislipidemia	0.642	0.192/2.148	0.472				
**Multivariate logistic regression analysis**							
DM	4.884	1.218/19.583	0.025*	J-CTO score	15.882	1.959/134.631	0.011*

Data are expressed as mean ± SD or number (%)

*BMI*: body mass index, *CKD*: chronic kidney disease, *DM*: diabetes mellitus, *G* (*N*): normal LVEF group, *G* (*M*): mid-range LVEF group, *G* (*L*): low LVEF group, *HTN*: hypertension, *:significant

**Fig. 1:**
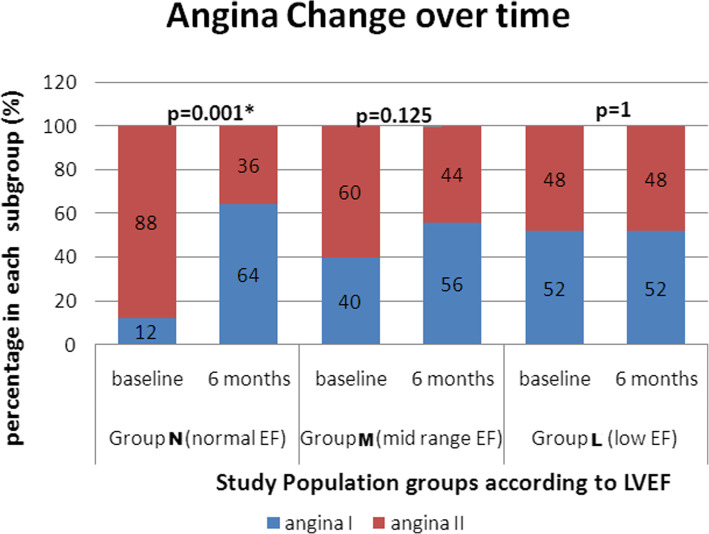
Change in angina class serially estimated (%) in the 3 study groups at baseline and 6 months post CTO PCI

Clinically, regarding angina follow up, each group was subdivided into 2 subgroups: angina I (patients with no angina or CCS class 1 or 2) and angina II (patients with CCS class ≥ 3). In group (N), there was a significant improvement in angina 6 months post PCI (McNemar = 0.01., *p* value < 0.001). Despite angina improvement was evident in the other 2 groups, yet without statistical significance (*p* > 0.05) (Fig. 1).

**Fig. 2:**
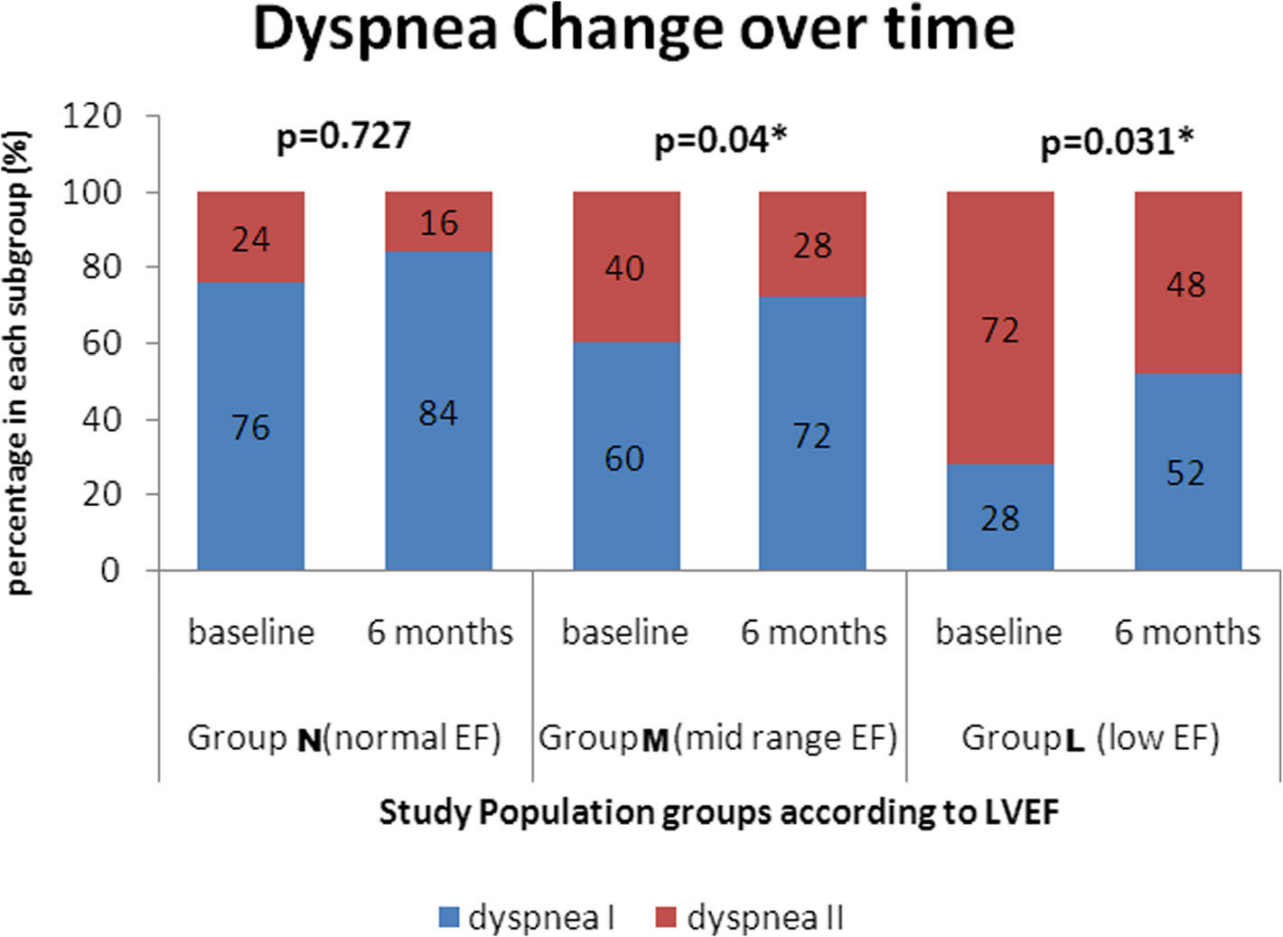
Change in dyspnea class serially estimated (%) in the 3 study groups at baseline and 6 months post CTO PCI

In the same context, each group was subdivided into 2 subgroups regarding dyspnea grades: dyspnea I (patients with no dyspnea or NYHA class 1 or 2) and dyspnea II (patients with NYHA class ≥ 3). There was a significant improvement in the grade of dypnea 6 months post CTO PCI, in group (M) (McNemar = 3.841, *p* value < 0.05) and group (L) (McNemar = 8.615, *p* value < 0.05) (Fig. 2).

**Fig. 3:**
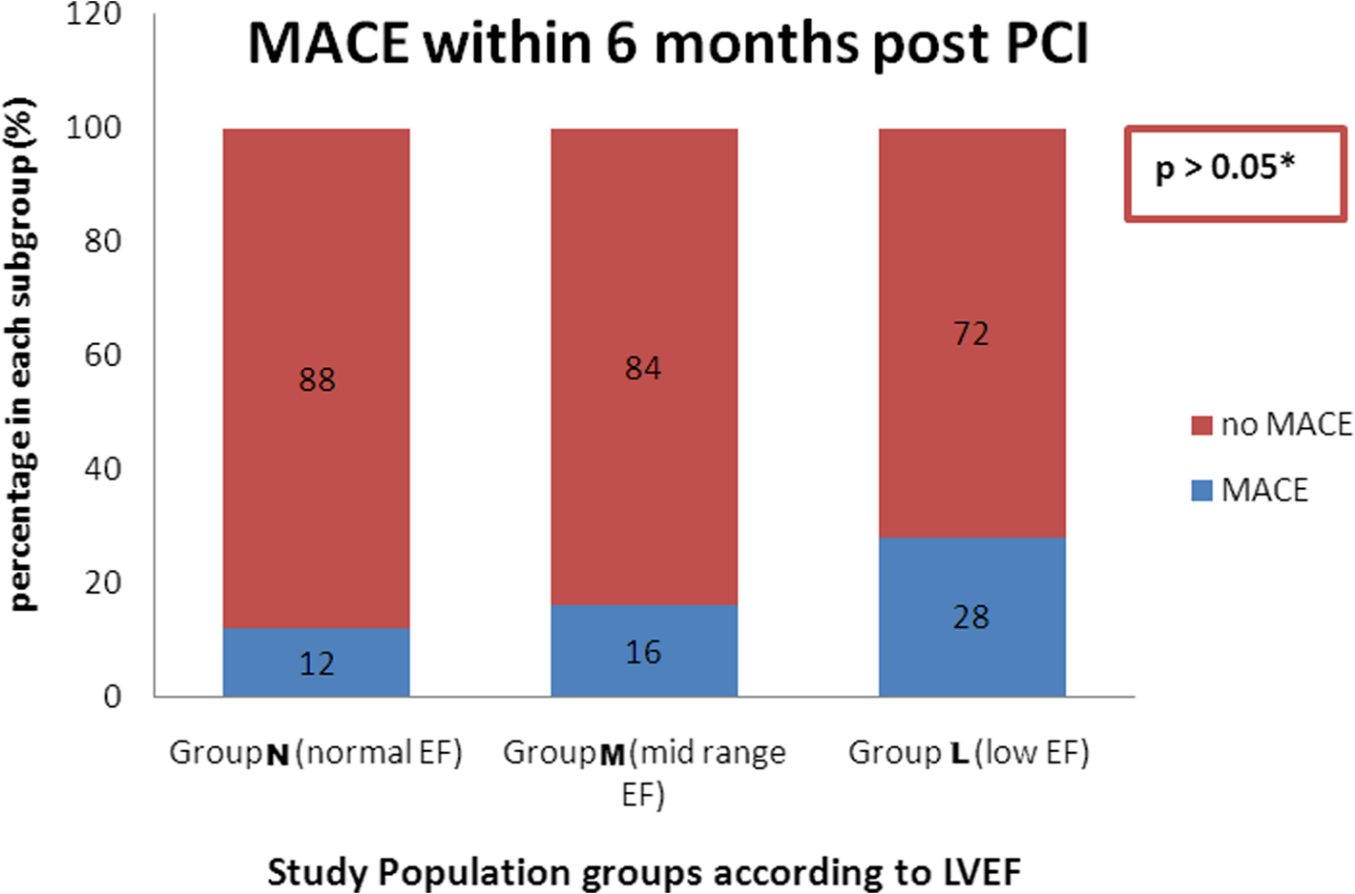
Major adverse cardiac events estimated (%) in the 3 study groups within 6 months post CTO PCI

All patients of the 3 study groups were assessed during the period of 6 months follow-up for MACCE. There was no mortality cases recorded in any group, whereas MACCE were recorded in 14 (18.6 %) out of 75 cases. In group (N), MACCE occurred in 3 (12%) of 25 patients (2 cases of NSTE-ACS with non-TVR and 1 case of stroke). In group (M), 4 (16%) of 25 patients had MACCE (2 cases of decompensated heart failure needing hospitalization, 1 case of non-fatal MI with non-TVR, and 1 case of TVR). In group (L), MACCE were recorded in 7 (28%) of 25 patients (4 cases of decompensated heart failure needing hospitalization, 1 case of non-fatal MI with non-TVR, 1 case of stroke and 1 case of malignant arrhythmia). There was no significant difference between the three groups regarding the reported MACCEs (*χ*^2^ = 2.283 and *p* > 0.05) (Fig. 3).

## Discussion

The benefit and safety of CTO PCI in patients with low and mid-range LVEF continue to be of limited evidence. Successful CTO PCI was claimed to be associated with the improvement of cardiovascular outcome in comparison with unsuccessful procedures [18, 19]. However, myocardial revascularization guidelines still did not address any solid indication for the most convenient management in patients with CTOs and impaired LVEF [7, 8].

Regarding baseline clinical characteristics, our data confirmed that the low LVEF group had more diabetic and CKD patients, compared with preserved and mid-range LVEF groups. Moreover, patients having low LVEF were more complaining of accelerated exertional dyspnea (NYHA ≥ 3) compared to the other 2 groups. This finding is in concordance with Galassi et al. [20] and Tajstra et al. [6], who exhibited a higher prevalence of DM, CKD and peripheral vascular disease (PVD) in patients with CTO and low LVEF. Moreover, Tajstra et al. suggested that the presence of CTO in patients with systolic heart failure was associated with significantly higher 12-month all-cause and cardiovascular deaths compared with patients without CTO. Actually, only 3.5% of patients of this study were affected by CTO PCI [6].

In our study, CTO PCI procedural success rate was 82.6% in all population, 76% in low LVEF group and 84% in mid-range LVEF group, with no significant increase of periprocedural complications when compared with normal LVEF group. Except for one case of coronary perforation with pericardial tamponade mandating pericardiocentesis, no patients with low or mid-range LVEFs suffered from severe periprocedural complications after CTO PCI. Moreover, impaired LVEF was not a predictor of CTO PCI periprocedural complications.

These results came hand in hand with Galassi et al. who revealed no significant correlation between impaired LVEF and periprocedural complications [20]. In the same context, Tajti et al. showed that despite CTO PCI in patients with < 35% LVEF had lower technical success when compared with other two groups with 35-49% LVEF and > 50% LVEF (83% vs. 88% and 89%, *p* = 0.03), yet with similar procedural success (82% vs. 85% and 86%, *p* = 0.17) and major in-hospital complication rates (3.7% vs. 2.9% and 2.4%, *p* = 0.41) [21]. Similar results were recently reported by Danek et al. [22] with a low rate of periprocedural complications.

Although the CTO PCI techniques used in patients with low LVEF were similar to those performed in those with preserved and mid-range LVEF, the lowest amount of contrast should be considered, as 50% of periprocedural complications recorded in this high-risk group were related to CIN. This could explain why contrast load was significantly lower in the low LVEF population of our study. Accordingly, our data propose that CTO PCI is a safe strategy when performed by experienced operators even in high-risk patients.

Successful CTO PCI was reported to confer benefit to patients over failed PCI [23]*.* Comparison of successful and failed CTO PCI done in a large meta-analysis showed a 44% reduction in mortality, a 55% reduction in residual angina and a 78% reduction in subsequent CABG in successful CTO PCI [24]*.* This mortality benefit can be attributed to many factors. Ischemia from the CTO territory may cause arrhythmic death. The presence of a CTO has been shown to be associated with higher rates of ventricular arrhythmia and all-cause death [25]. Furthermore, patients with CTOs cannot withstand an acute MI as multiple territories are simultaneously affected, what so-called double jeopardy [26]*.*

It is generally accepted by consensus and guidelines that CTO PCI is indicated for symptomatic patients [27]*.* Improvement in angina status after CTO-PCI has also been shown in the previous studies in patients with preserved LVEF [28, 29]. In the current study, a good clinical outcome was achieved in the 3 groups and impaired LVEF was not associated with increased incidence of MACCE at 6 months follow up. Moreover, there was a significant improvement in NYHA functional class in patients with low and mid-range LVEF, with significant improvement of CCS class in patients with preserved LVEF.

These results are concordant with Galassi and his colleagues, as low LVEF did not independently predict MACCE at mid-term follow-up. There was also a significantly less dyspnea in patients with low LVEF and less angina in those with preserved LVEF 6 months post CTO PCI, with mid-range LVEF population being still in the gray zone [20]

Another finding, from a clinical point of view; showed an improvement in angina and NYHA functional class, after CTO recanalization in patients with HFrEF, which could lead to improved prognosis in this population and provide a rationale for attempting CTO recanalization after viability and/or ischemia confirmation in the CTO territory [30]*.*

Moreover, in a study by Borgia et al, the Seattle Angina Questionnaire-UK version (SAQ-UK) was used to assess patients’ angina quality of life after CTO PCI attempt. Compared to the unsuccessful group, the successful CTO PCI group reported fewer angina frequency and less physical limitation, and most were either asymptomatic or only CCS I [31]. Furthermore, Erdogan et al. showed that significant improvement of angina was achieved by successful CTO revascularization, where angina of CCS 2 or greater was improved in all patients who suffered from those degrees of angina prior to revascularization [32].

Needless to say that demonstration of myocardial viability is essential for improvement of survival and symptoms in patients with left ventricular dysfunction undergoing PCI regardless of the type of lesion [33].

Finally, what is really interesting in our study is being—up to our knowledge—the first study to evaluate the procedural consequences and clinical outcome post CTO PCI in such a peculiar category of mid-range LVEF (40-49%). Our study showed significant improvement in symptoms 6 months post PCI with no significant increase in periprocedural or mid-term adverse events, compared to patients with low or preserved LVEFs. In fact, further studies are necessary to address clinical characteristics, procedural details and more long term outcomes in such specific category as well defined by current guidelines.

## Conclusion

According to our study, CTO PCI represents an efficient and safe strategy in patients with mid-range LVEF (40-49%) and low LVEF (< 40%) affected by CTOs. In a 6 months follow-up period, significant improvement of mid-term clinical outcome, particularly dyspnea, was related to successful CTO PCI. Also, impaired LVEF categories were not associated with a higher risk of MACCE at follow-up.

## Limitations

The first limitation of our study is being non-randomized study; therefore, changes in clinical parameters could not be specifically confirmed if being due to successful CTO PCI alone or related to optimal medical therapy. Second drawback was the relatively small sample size. Third, the short term follow-up period may alter the proper assessment of clinical outcomes, especially in those with impaired LVEF. Fourth, we did not perform a second angiography at 6 months after PCI in any of the patients. Finally, our study did not include CABG in patients with low and mid-range LVEFs for comparison versus PCI.

## Data Availability

Available by the corresponding author upon request.

## Abbreviations

2-D: Two dimensional
ACS: Acute coronary syndrome
AF: Atrial fibrillation
BMI: Body mass index
CABG: Coronary artery bypass grafting
CAD: Coronary artery disease
CCS: Canadian Cardiovascular Society
CIN: Contrast-induced nephropathy
CKD: Chronic kidney disease
CTO: Chronic total occlusion
DM: Diabetes mellitus
DSE: Dobutamine stress echocardiography
HFrEF: Heart failure with reduced ejection fraction
IHD: Ischemic heart disease
LVEF: Left ventricular ejection fraction
MACCE: Major adverse cardiac and cerebrovascular events
Nom.: Number
MI: Myocardial infarction
NYHA: New York Heart Association
PCI: Percutanous coronary intervention
PVD: Peripheral vascular disease
SAQ-UK: Seattle Angina Questionnaire-UK version
SD: Standard deviation
SPSS: Statistical Package for the Social Sciences
TIMI: Thrombolysis in myocardial infarction
TVR: Target vessel revascularization
